# A case report of a 40-year-old woman with endomyocardial fibrosis in a non-tropical area: from initial presentation to high urgent heart transplantation

**DOI:** 10.1186/s12872-019-1243-8

**Published:** 2019-12-19

**Authors:** Gernot Wagner, Markus Haumer, Gerhard Poelzl, Dominik Wiedemann, Andreas Kliegel, Robert Ullrich, Gerald Gartlehner, Andreas Zuckermann, Ludwig Müller, Harald Mayr, Deddo Moertl

**Affiliations:** 1 Department for Evidence-based Medicine and Evaluation, Danube University Krems, Dr. Karl Dorrek Strasse 30, 3500 Krems, Austria; 2 Department of Internal Medicine 2, Landesklinikum Wiener Neustadt, Corvinusring 3-5, 2700 Wiener Neustadt, Austria; 3 Department of Internal Medicine III, Clinical Division of Cardiology & Angiology, Innsbruck Medical University, Anichstrasse 35, 6020 Innsbruck, Austria; 4 Department of Cardiac Surgery, Medical University Vienna, Waehringer Guertel 18-20, 1090 Vienna, Austria; 5 Department of Internal Medicine 3, University Hospital St. Poelten, Karl Landsteiner University of Health Sciences, Dunantplatz 1, 3100 St. Poelten, Austria; 6 Institute for Research of Ischaemic Cardiac Disease and Rhythmology, Karl Landsteiner Society, Propst-Fuehrer-Strasse 4, 3100 St. Poelten, Austria; 7 Clinical Institute of Pathology, Medical University Vienna, Waehringer Guertel 18-20, 1090 Vienna, Austria; 8 RTI International, 3040 East Cornwallis Road, PO Box 12194, Research Triangle Park, North Carolina 27709-2194 USA; 9 Department of Cardiac Surgery, Medical University Innsbruck, Anichstrasse 35, 6020 Innsbruck, Austria

**Keywords:** Restrictive cardiomyopathy, Endomyocardial fibrosis, Heart failure, Eosinophilia

## Abstract

**Background:**

Endomyocardial fibrosis (EMF) represents the most common cause of restrictive cardiomyopathy worldwide. Despite a high prevalence in tropical regions, it occasionally occurs in patients who have never visited these areas. While researches have proposed various possible triggers for EMF, etiology and pathogenesis remain largely unknown. Diagnosis is based on patient history, heart failure symptoms, and echocardiographic signs of restrictive ventricular filling, atrioventricular valve regurgitation and frequently apical thrombus. Following is a case report of an Austrian patient with EMF who eventually had to undergo a heart transplant. This case report strives to promote awareness for this in non-tropical areas uncommon but nevertheless detrimental disease.

**Case presentation:**

A 40-year-old woman was presented at our emergency department with chest pain and fever up to 38.1° Celsius. Plasma troponin-T levels and inflammatory markers were slightly elevated, but the echocardiogram was without pathological findings. The patient was hospitalized on the suspicion of acute myocarditis and discharged soon after improvement. Eight months later, she was presented again with chest pain and symptoms of heart failure. The echocardiogram showed normal systolic left ventricular (LV) function with LV wall thickening and severe restrictive mitral regurgitation as well as aortic and tricuspid regurgitation. Coronary angiogram was normal but right heart catheterization showed pulmonary hypertension due to left heart disease. Further diagnostic workup with cardiac magnetic resonance imaging revealed subendocardial late enhancement and apical thrombus formation in the left ventricle compatible with the diagnosis of EMF. A comprehensive diagnostic workup showed no evidence of infection, systemic immunologic or hematological disease, in particular hypereosinophilic syndrome. After a multidisciplinary consideration of several therapeutic options, the patient was listed for heart transplantation. On the waiting list, she deteriorated rapidly due to progressive heart failure and finally underwent a heart transplantation. Histological examination confirmed the diagnosis of EMF. Six years after her heart transplantation, the patient was presented in an excellent clinical condition.

**Conclusions:**

Even in non-tropical regions, the diagnosis of EMF should always be considered in restrictive cardiomyopathy. Knowledge of the distinct phenotype of EMF facilitates diagnosis, but comprehensive workup and therapeutic management remain challenging and require a multidisciplinary approach.

## Background

Endomyocardial fibrosis (EMF) is characterized by subendocardial fibrosis primarily of the apex and inflow tracts of the right ventricle, left ventricle, or both [1]. The clinical manifestation of this disease is determined by restriction of left ventricular filling and atrioventricular valve regurgitation due to involvement of papillary muscles and chordae tendinae, ultimately resulting in heart failure [2].

First described by Davies in Uganda in 1948 [3], EMF is considered the most common cause of restrictive cardiomyopathy worldwide with a high regional variety of prevalence [2]. While there is a high prevalence of EMF in tropical regions such as Brazil, south India, Uganda, Nigeria and the Ivory Coast, with a population prevalence of up to 20% in Mozambique [4], there are only anecdotal reports from non-tropical regions [5, 6]. Data from Uganda describe a peak at ages 10 and 30 [7].

Even though several causes such as eosinophilia, infections, autoimmunity, genetic predisposition, dietary and environmental factors have been proposed, etiology and pathogenesis remain largely unknown [2]. A cardiotoxic effect of eosinophil granulocytes is often discussed as a major pathogenetic contributor [8].

The diagnosis of EMF is mainly based on patient history, as well as clinical and echocardiographic findings [9]. Echocardiography typically is presented with a restrictive filling pattern, apical fibrosis of one or both ventricles and tethering of atrioventricular valves, leading to mitral and/or tricuspid regurgitation, and giant atrial enlargement. Apical thrombi are frequent. In contrast to other causes of apical thrombi, for example myocardial infarction, the apex maintains its contractile function in EMF. Cardiac catheterization is not obligatory to confirm diagnosis of EMF, however, invasive hemodynamic examination would indicate a restrictive pattern with the typical diastolic dip-and-plateau sign of the ventricular pressure curves.

Cardiac magnetic resonance (CMR) imaging shows subendocardial late enhancement and possibly apical thrombus formations [10, 11]. CMR could help to select patients who may benefit from steroid therapy in early stages of the disease with active inflammation but is of limited availability in most endemic regions.

EMF is sometimes indistinguishable from late stage Loeffler’s endocarditis [12]. However, Loeffler’s endocarditis is primarily observed in temperate climates and associated with marked hypereosinophilia, thromboembolic events, and systemic arteritis. Direct cardiotoxic effects of eosinophils lead to necrosis, thrombus formation and finally fibrosis [13]. The similarities between late stage Loeffler’s endocarditis and EMF suggest common pathophysiological processes in at least a subgroup of EMF patients.

Since the natural course of the disease is not well understood and reliable evidence to support therapeutic decisions is lacking, medical therapy aims primarily to achieve symptom relief and prevent sequelae of the condition. Diuretics are given for fluid retention, heart rate lowering drugs for rate control in atrial fibrillation, and anticoagulation in cases of a ventricular thrombus and/or atrial fibrillation [9]. Immunosuppressive therapy might be considered in early stages of the disease with still predominant acute myocarditis [2, 9]. However, patients with EMF usually appear at a stage when active myocarditis has been replaced by substantial fibrosis and immunosuppression would not be efficacious anymore. At this late stage, limited data suggest that endomyocardial resection with valve replacement or repair might be a treatment option, [2, 14, 15] albeit with high perioperative mortality of up to 20% [2, 14, 16, 17].

We report on a rare case of EMF diagnosed in Austria initially diagnosed as rapidly resolving, unspecific myocarditis, which finally underwent heart transplantation. The aim of this case report is to promote awareness for EMF as a potential cause of restrictive cardiomyopathy even in non-tropical regions and discuss the challenging interdisciplinary diagnostic workup and management.

## Case presentation

In January 2011, a 40-year-old woman was presented at our emergency department with a six-day history of fever up to 38.1 degrees Celsius every evening and with exertional retrosternal chest pain. She reported no other symptoms like dyspnea, abdominal pain, urinary, or stool abnormalities. Past medical history revealed an appendectomy at the age of 12, a caesarean section in 1993 and a thyroidectomy in November 2010 due to Graves’ disease. Approximately 5 months prior to presentation (August 2010), the patient had been on holiday in Hurghada, Egypt. Her long-term medication included levothyroxin, esomeprazole, and tizanidin. Her primary care physician had prescribed amoxicillin/clavulanic acid the day before presentation at our department. The patient worked as a nurse in a nursing home for the elderly. She smoked 15 cigarettes per day (15 pack years), occasionally drank alcohol, and had an allergy to house dust mites. Her cardiovascular risk factors were smoking, hypercholesterolemia, and a family history of myocardial infarction.

The patient was in a good general condition. On arrival, temperature was 36.9° Celsius, blood pressure 150 over 90 mmHg, heart rate 130 beats per minute, and oxygen saturation 97% with ambient air. Auscultation and percussion of lung and heart were unremarkable. The abdomen showed no resistance or tenderness. No peripheral edema was observed. Results of a basic neurological examination were unremarkable and the skin was normal.

An electrocardiogram (ECG) revealed sinus tachycardia with a heart rate of 120 beats per minute, normal axis, and slight T-wave inversion in leads V3-V6. The white blood cell count was 13.2 G/l with 4% (0.5 G/l) eosinophils, hemoglobin 13.6 g/dl, platelet count 176 G/l. Levels of serum creatinine, blood urea nitrogen and electrolytes were normal. The concentrations of gamma glutamyltransferase (54 U/l) and alkaline phosphatase (122 U/l) were slightly elevated. C-reactive protein (CRP, 6.11 mg/dl), high sensitive Troponin-T (hs TnT, 67 pg/ml) and D-Dimer (2.06 mg/l) were also elevated (Table 1). Chest x-ray was unremarkable and computed tomography angiography showed no signs of pulmonary embolism. Transthoracic echocardiogram was without pathological findings. In particular, it revealed normal right and left ventricular function, no wall motion abnormalities and no evidence of valve disease or pericardial effusion. Based on the history, laboratory result and imaging findings, (peri-) myocarditis was suspected and the patient was admitted for observation and symptomatic treatment with aminosalicylic acid and ibuprofen.

**Table 1:** Laboratory findings

	Reference	18.01.2011	30.09.2011	30.11.2011	20.03.2012
	Range^a^	First Presentation	Suspected EMF	Follow-up visit	Listing for HTX
Hemoglobin (g/dl)	12.0–16.0	13.6	12.4	**10.7**	12.2
White blood-cell count (G/l)	4.0–10.0	**13.2**	**14.7**	7.4	**12.8**
Differential count (G/l - %)					
Neutrophils	2.0–7.0/50–70	8.0/61	**10.8/74**	4.4/60	**9.3/72**
Eosinophils	< 0.4/< 4	**0.5/4**	**0.8/6**	0.3/4	0.1/1
Basophils	< 0.2/< 1	0/0	0/0	0/0	0/0
Monocytes	0.2–1.0/2–10	1.0/7	0.5/4	0.6/8	0.8/0
Lymphocytes	0.8–4.0/20–40	3.7/28	2.5/17	2.1/29	2.6/20
Platelet count (G/l - %)	150–450	176	180	314	310
Sodium (mmol/l)	136–145	140	141	141	138
Potassium (mmol/l)	3.50–5.10	4.03	4.86	4.64	4.51
Creatinine (mg/dl)	0.50–0.90	0.79	**1.17**	**0.95**	**1.23**
BUN (mg/dl)	6.0–20.0	10.7	16.6	12.3	**23.5**
NT-proBNP (pg/ml)	< 125	**1501**	**3697**	**1595**	**2054**
LDH (U/l)	135–214	**261**	**375**	158	160
C-reactive protein (mg/dl)	< 0.50	**6.11**	**1.47**	**2.27**	0.38
TnT-hs (pg/ml)	< 14	**67**	7	3	3

Abbreviations: *BUN* Blood urea nitrogen, *EMF* Endomyocardial fibrosis, *HTX* Heart transplantation, *LDH* Lactate dehydrogenase, *NT-proBNP* N-terminal pro-B-type natriuretic peptide, *TnT-hs* Troponin T high sensitive

^a^ Reference Range for Adults, Department for Laboratory Medicine, University Hospital St. Poelten, Austria

Bold letters indicate values outside of the normal range

On day two of hospitalization, the patient had no more complaints, cardiac enzymes and CRP decreased, and on day three the patient was discharged. Further diagnostic procedures included blood culture, immunology (anti-nuclear antibodies with subsets, anti-neutrophil cytoplasmic antibodies, [ANCA]), virus serology for typical cardiotropic viruses, antibodies (complement-fixation test) against mycoplasma, coxiella burnetii (Q-fever), chlamydia psittaci (ornithosis) and interferon-γ release assays were negative, except for perinuclear anti-neutrophil cytoplasmic antibodies (p-ANCA) which were elevated to 47 U/ml (0–20).

Eight months later, in September 2011, the patient was re-hospitalized with chest pain for 1 week, dyspnea on minimal exertion (New York Heart Association [NYHA] functional class IV), which had developed within 4 days, and a new echocardiographic finding of severe mitral regurgitation. Medication comprised levothyroxin, bisoprolol and simvastatin. On examination, the temperature was 36.9° Celsius, blood pressure 100 over 75 mmHg, heart rate 99 beats per minute and oxygen saturation 97% (pulse oximetry) with ambient air. Auscultation and percussion of lung and heart were unremarkable and the abdomen showed no resistance or tenderness. No peripheral edema was observed. Laboratory results showed elevated white blood-cell count 14.7 G/l with 6% (0.8 G/l) eosinophilic, NT-proBNP (3697 pg/ml), LDH (375 U/l) and CRP (1.47 mg/dl). Hs-TnT and creatine kinase were normal.

On ECG a new P-mitrale was detected (see Additional file 1). Transthoracic and transesophageal echocardiogram showed a hyperdynamic left ventricle with preserved left ventricular ejection fraction without any regional wall motion abnormalities and dilated left and right atria. The left ventricular apical and lateral wall were thickened while the interventricular septum was normal. Doppler recordings of mitral valve inflow showed a restrictive filling pattern with severe mitral regurgitation (see Additional file 2). Further aortic and tricuspid regurgitation as well as significant elevated systolic pulmonary artery pressure were observed in the absence of pericardial effusion.

Coronary angiography was unremarkable but invasive hemodynamic evaluation showed postcapillary pulmonary hypertension (mean pulmonary artery pressure 42 mmHg) with markedly elevated left ventricular filling pressures (LV end-diastolic pressure 39 mmHg) and reduced cardiac index (1.74 L/min/m^2^). Left ventriculography showed apical contrast dye sparing (see Additional files 3, 4 and 5).

**Fig. 1:**
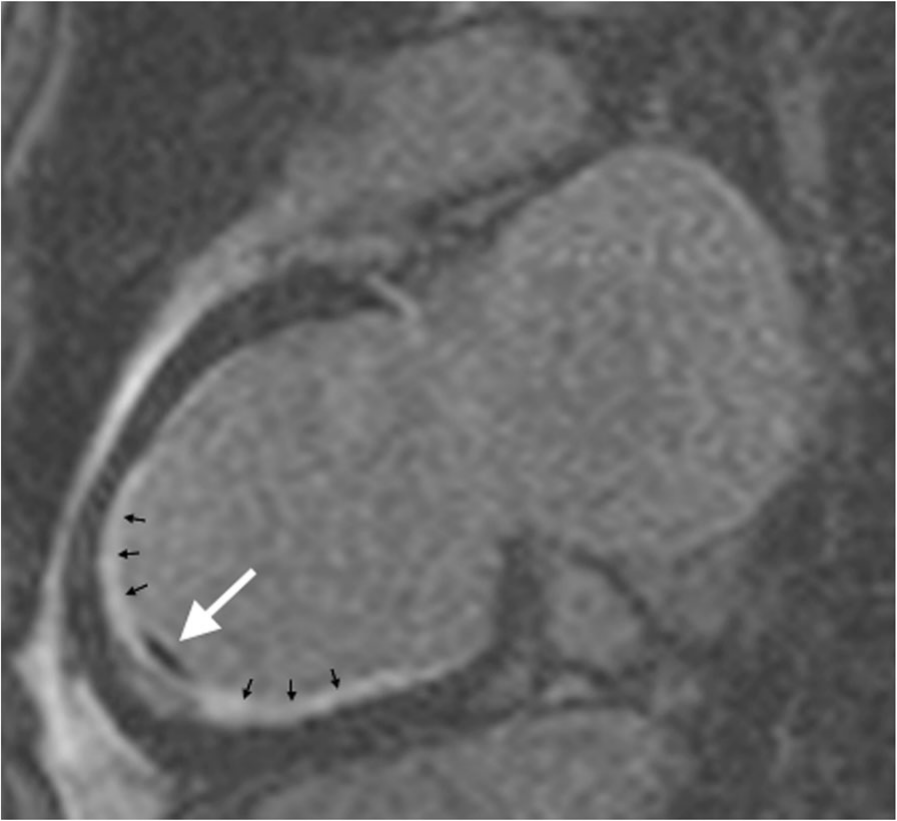
Cardiac magnetic resonance imaging (1.5 Tesla, 2 chamber view, late gadolinium enhancement, inversion recovery, inversion time [TI]: 190 ms). Cardiac magnetic resonance imaging showing extended semicircumferential subendocardial late enhancement indicating endomyocardial fibrosis (small black arrows) and an apical thrombus (long white arrow)

CMR imaging confirmed severe mitral regurgitation and revealed a mildly dilated left ventricle with a left ventricular ejection fraction of 45% and an apical thrombus. Extended semicircumferential subendocardial late enhancement with partial involvement of the papillary muscles was compatible with EMF (see Fig. 1).

Enalapril and anticoagulation with enoxaparin followed by phenprocoumon were started for heart failure and the apical thrombus, respectively, and the patient was discharged in stable condition. For a second opinion and the development of a management plan, the patient was referred to a university heart failure unit with a focus on rare cardiomyopathies in November 2011. Endomyocardial biopsy was planned from the right ventricle - to avoid left ventricular thrombus mobilization – but was hampered by the dense consistency of the fibrotic endocardium. After several attempts the right ventricular free wall was perforated resulting in pericardial tamponade, which was immediately treated successfully by pericardiocentesis and drainage. In synopsis of all findings, the patient was given the diagnosis of EMF without another attempt for histological confirmation.

Further workup revealed no evidence of infection, systemic immunologic, hematological, or solid malignant disease. Microscopy on helminthic eggs or intestinal protozoa was negative. The patient progressively deteriorated with decreasing walking distances of < 100 m, semiorthopnoea, and increasing signs of congestion so that a rapid therapeutic decision was necessary. The interdisciplinary heart team, consisting of cardiologists and cardiac surgeons, discussed several therapeutic options and decided to evaluate and list the patient for heart transplantation.

**Fig. 2:**
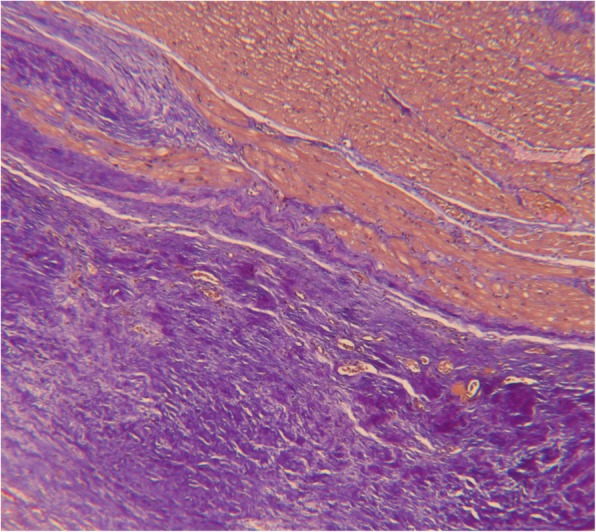
Histology of explanted heart. Image shows broad fibrosis of the endocardium and myocardium with increased vascularization, fibroblasts, chronic inflammatory infiltration (few mast cells and eosinophilic cells) as well as in particular subendocardial interstitial fibrosis

**Fig. 3:**
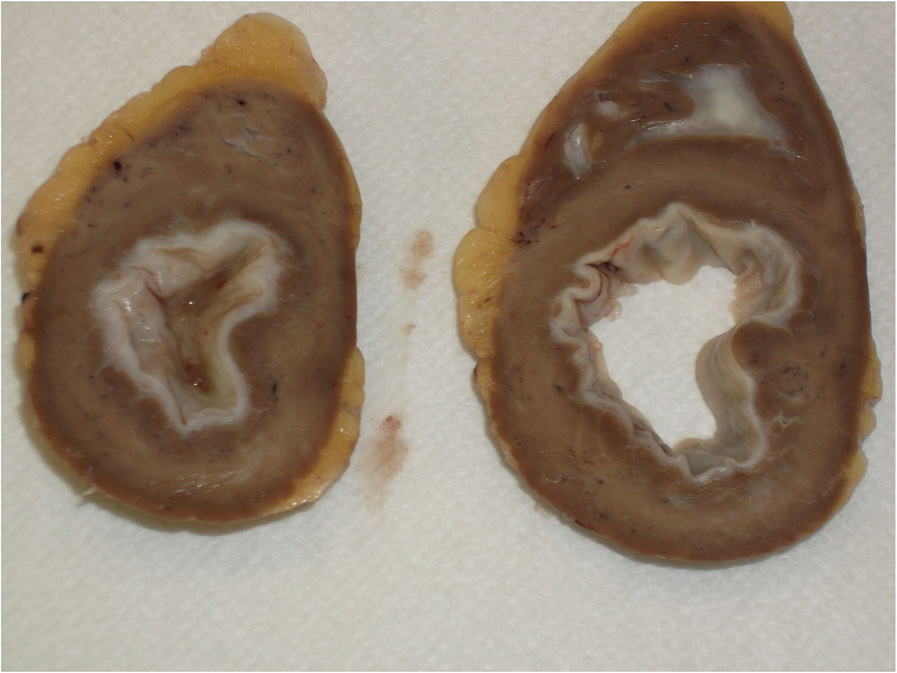
Macroscopic image of explanted heart. Image shows severe fibrosis of the endocardium involving both ventricles

While on the waiting list, the patient’s condition initially stabilized with enalapril, bisoprolol, and furosemide. Due to continuous progression of heart failure starting in June 2012 she underwent high urgency heart transplantation in August 2012. Macroscopic and microscopic findings of the explanted heart confirmed the diagnosis of EMF (see Figs. 2 and 3). Both ventricles revealed severe fibrosis of the endocardium with apical predominance. The neighboring myocardium was characterized by hypervascularization, fibroblasts, chronic inflammatory infiltration with few mast cells and eosinophils as well as interstitial fibrosis in subendocardial layers. Six years after heart transplantation the patient was in an excellent clinical condition (see Table 2).

**Table 2:** Timeline of patient’s history from first presentation until end of follow-up

Year	Month	History
2010	August	• Holiday in Hurghada, Egypt
2011	January	• First presentation at emergency department with fever and exertional retrosternal chest pain • Discharge after 3 days without complaints • Suspected (Peri-)myocarditis
	September	• Re-hospitalization with chest pain for 1 week, dyspnea on minimal exertion (NYHA IV) • Comprehensive diagnostic work-up • Medical treatment for heart failure and anticoagulation for apical thrombus were started • Discharge in stable condition • Suspected EMF
	November	• Referred to a university heart failure unit with a focus on rare cardiomyopathies for second opinion • Unsuccessful endomyocardial biopsy • Diagnosis of EMF without another attempt for histological confirmation
2012	June	• Continuous progression of heart failure
	August	• High urgency heart transplantation
2018		• Excellent outcome after heart transplantation

Abbreviations: *EMF* Endomyocardial fibrosis, *NYHA* New York Heart Association functional class

## Discussion

This patient with the rare diagnosis of EMF outside tropical regions [18], initially was presented with unspecific findings of acute myocarditis including fever, chest pain, electrocardiographic abnormalities, elevated markers of inflammation and cardiomyocyte damage. While no clear evidence of EMF was detectable at that moment, this was obviously the initiation of the disease.

EMF was confirmed histologically only after heart transplantation, so that the patient had to be diagnosed upon clinical, imaging and laboratory findings. While most findings were supportive of EMF - especially the restrictive phenotype due to fibrotic remodeling in echocardiography and CMR involving primarily the apex, the subsequent atrioventricular valve regurgitation and the apical thrombus - the etiology and the definite differentiation from Loeffler’s endocarditis needs to be discussed.

In our patient, we observed mild eosinophilia at two acute hospitalizations (0.5 G/l and 0.8 G/l) with a time interval of 8 months. This is compatible with EMF [19], while Loeffler’s endocarditis usually is associated with eosinophilia of > 1.5 G/L for at least 6 months. However, we cannot rule out that transient higher levels of eosinophilia have been missed between the two time points. Eosinophilia is not a consistent finding in EMF [20]. In a case series of 15 patients with EMF from rural Egypt, all patients had a history of infection with Schistosoma but none had eosinophilia [6], which suggests that a complete etiological workup is necessary in EMF independent of the presence of eosinophilia. Although her recent stay in Egypt raised the probability for parasitic infection, this could be excluded after comprehensive testing. Our workup did not reveal any other diseases typically associated with eosinophilia and/or EMF including eosinophilic granulomatosis with polyangiitis (EGPA, formerly designated Churg-Strauss syndrome), hypersensitivity reactions, which are usually medication induced, and malignancy like leukemia [13]. Therefore, the trigger of eosinophilia remained unclear. Since eosinophilia was transient, the patient’s allergy to house dust mite is an unlikely explanation.

The role of the p-ANCA in this patient remains unclear. The combination of positive p-ANCA and eosinophilia are typical for EGPA, which was – however – excluded in the absence of vasculitis [21]. The presence of ANCA with or without vasculitis has also been associated with antithyroid drugs, even many years after exposure [22]. Therefore, the presence of p-ANCA might be related to our patient’s history of Grave’s disease and/or thiamazol intake until thyroidectomy.

With the exclusion of parasitic infection, hypereosinophilic syndrome, malignant disease, vasculitis or connective tissue disease and other factors that are associated with EMF in tropical regions like malnutrition or cassava intake, the underlying trigger for EMF in our patient remained unclear.

Immunosuppressive therapy may be a treatment option in early stages of EMF [9, 20], but advanced stages of the disease such as in our patient usually requires cardiac surgery. However, due to the extended biventricular, bivalvular involvement and the reported perioperative mortality of up to 20% [14, 23], the risk for an adverse outcome of endocardial decortication and atrioventricular valve replacement was considered as unacceptably high. An unfavorable prognosis of this patient was predicted from the severity of symptoms, biventricular involvement, atrioventricular valve regurgitation, and pulmonary hypertension, which was confirmed by the subsequent rapid deterioration and frequent heart failure hospitalizations. Therefore, listing for heart transplantation was regarded as the best treatment option for this patient. The excellent outcome 6 years after heart transplantation reaffirmed this treatment decision.

## Conclusion

In conclusion, EMF should be considered for differential diagnosis in patients with restrictive cardiomyopathy even in non-tropical areas. Diagnostic workup of patients with EMF is challenging but increased awareness of the disease in non-endemic regions might lead to detection of EMF at earlier stages where immunosuppressive therapy might still significantly modify the course of the disease. The often proposed surgical treatment with decortication and valve surgery has a high mortality. Despite only anecdotal evidence [24, 25], in selected patients heart transplantation seems a good option where available.

## Supplementary information

**Additional file 1.** ECG.

**Additional file 2.** Echocardiographic images.

**Additional file 3.** Loop of left ventriculography, left anterior oblique (LAO) view.

**Additional file 4.** Loop of left ventriculography, right anterior oblique (RAO) view.

**Additional file 5.** Still image of left ventriculography, right anterior oblique (RAO) view.

## Data Availability

Since this is a case report, data sharing is not applicable to this article as no datasets were generated or analysed.

## Abbreviations

ANCA: Anti-neutrophil cytoplasmic antibodies
CMR: Cardiac magnetic resonance
CRP: C-reactive protein
ECG: Electrocardiogram
EGPA: Eosinophilic granulomatosis with polyangiitis
EMF: Endomyocardial fibrosis
hs-TnT: High sensitive troponin T
LV: Left ventricular
NT-proBNP: N-terminal pro-B-type natriuretic peptide
NYHA: New York Heart Association
p-ANCA: Perinuclear anti-neutrophil cytoplasmic antibodies
