# Supplementary material for: A case report of a 40-year-old woman with endomyocardial fibrosis in a non-tropical area: from initial presentation to high urgent heart transplantation
Source: BMC Cardiovasc Disord. 2019 Dec 19;19:302. doi: 10.1186/s12872-019-1243-8 (PMC6933894; doi:10.1186/s12872-019-1243-8)
Supplement: Supplementary file 1 — Additional file 1. ECG. [file 12872_2019_1243_MOESM1_ESM.pdf]

Additional file 2. ECG

HF 98/min

Vermessungsergebnisse:

QRS	:		86 ms
QT/QTcB	:	350 /	449 ms
PQ	:		132 ms
P	:		106 ms
RR/PP	:	608 /	620 ms
P/QRS/T	:	80/ 60/	-75 Grad
QTD/QTcBD	:	70 /	90 ms
Sokolow	:		1.6 mU
NK	:		14

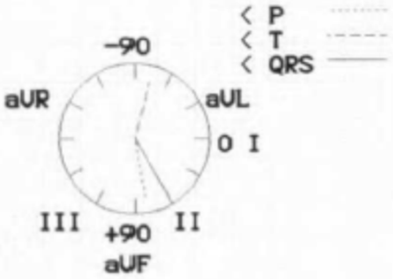

unbestätigter Bericht.

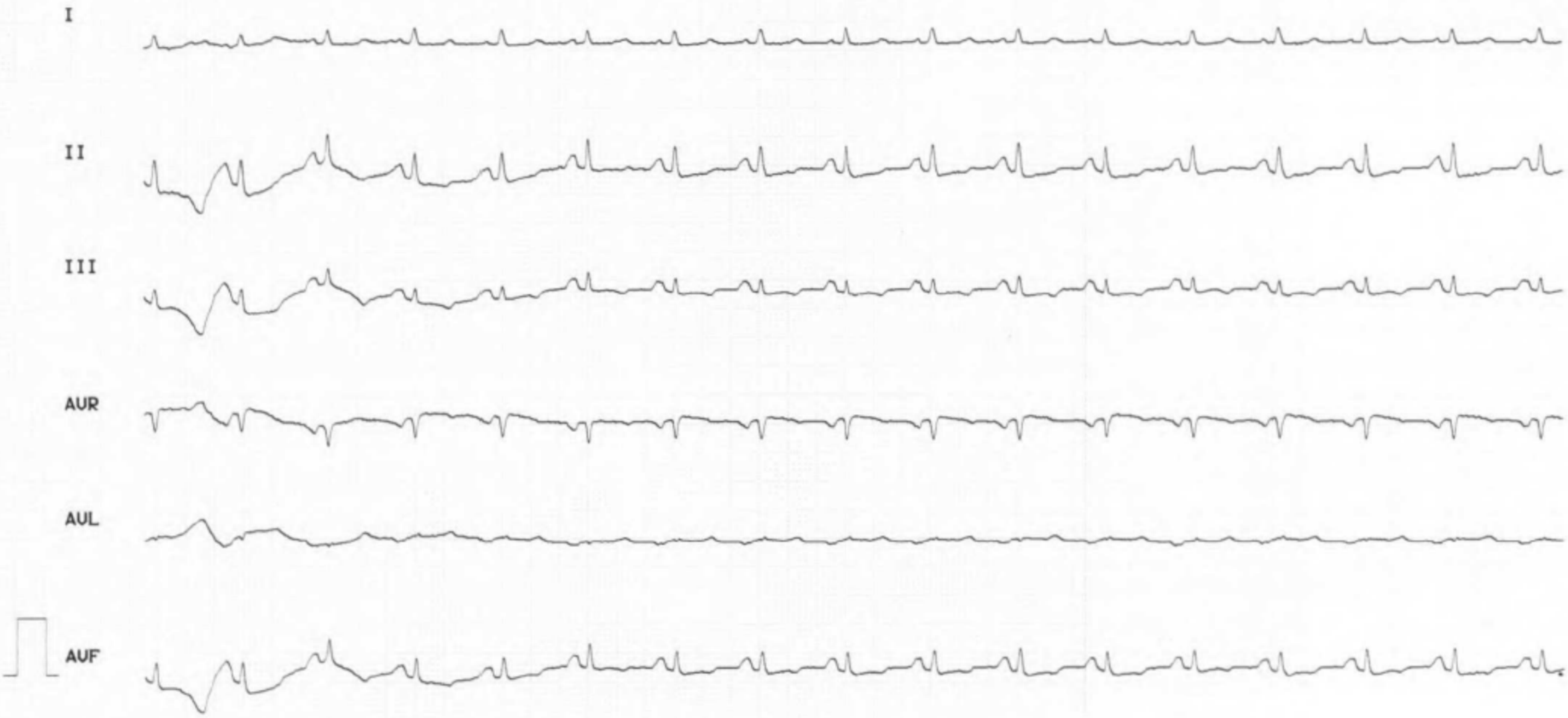

[redacted] 30. Sep. 2011 15:08:16 25mm/s 10mm/mU ADS 50Hz 0.08 - 20Hz 6_F2_25 Automatik U5.21 12i (1)

GE Medical Systems IT HELLIGE CONTRAST ® 226 166 11 CE D 777

GEMS IT MAC1200 ST , LKH.ST.PÖLTEN 3.MED NFA

HF 98/min

unbestätigter Bericht.

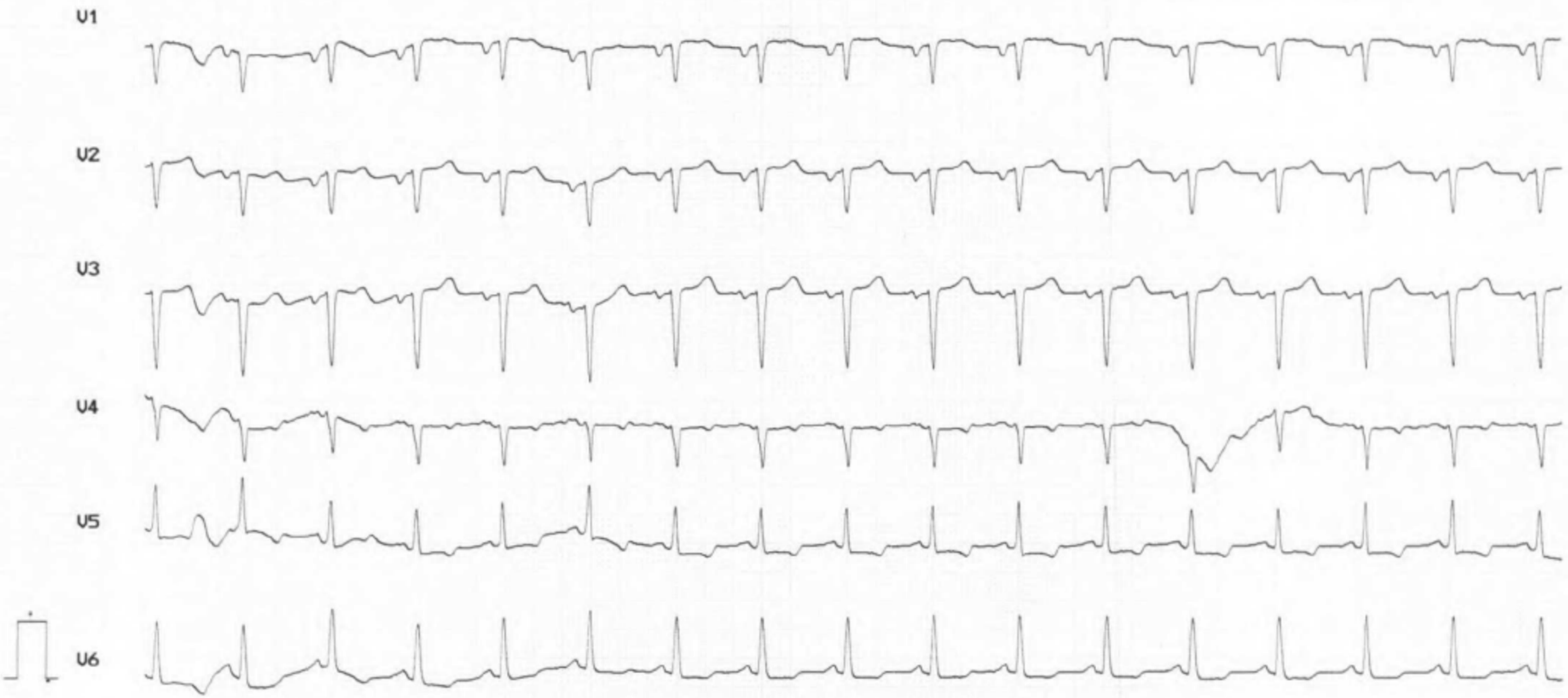

[redacted] 30.Sep.2011 15:08:16 25mm/s 10mm/mV ADS 50Hz 0.08 - 20Hz 6_F2_25 Automatik V5.21 12i (1)
