# Supplementary material for: A case report of a 40-year-old woman with endomyocardial fibrosis in a non-tropical area: from initial presentation to high urgent heart transplantation
Source: BMC Cardiovasc Disord. 2019 Dec 19;19:302. doi: 10.1186/s12872-019-1243-8 (PMC6933894; doi:10.1186/s12872-019-1243-8)

Additional file 3.

Echocardiographic images from November 2011 showing
(A) left atrial enlargement,
(B) severe mitral regurgitation, and
(C) apical endomyocardial fibrosis

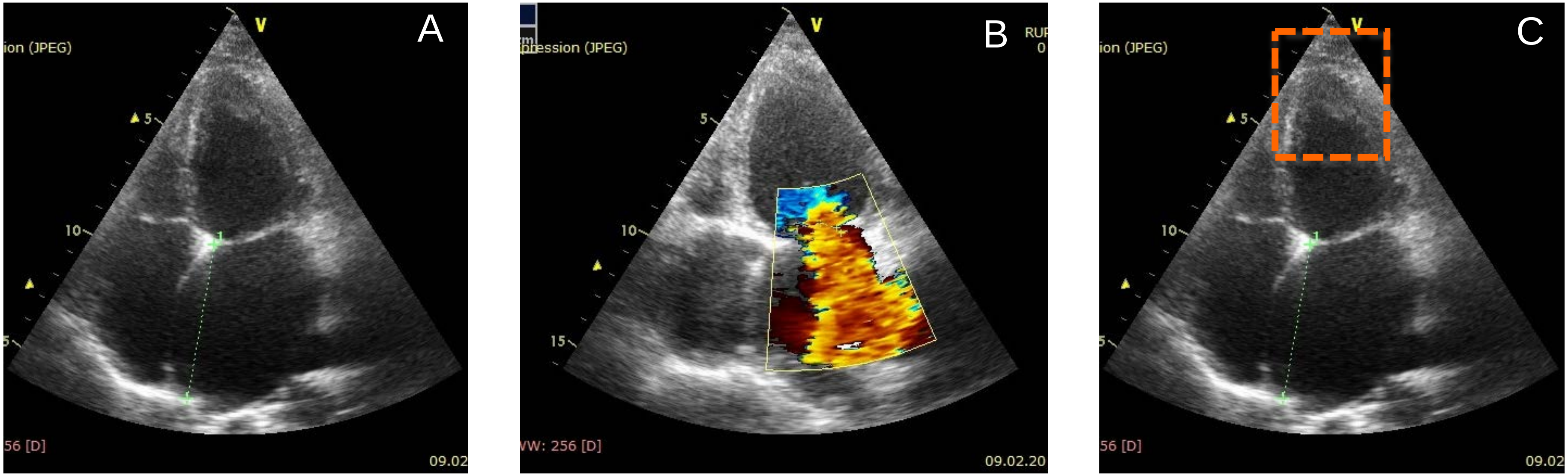

Supplement: Supplementary file 2 — Additional file 2. Echocardiographic images. [file 12872_2019_1243_MOESM2_ESM.pdf]
