# Supplementary material for: A case report of a 40-year-old woman with endomyocardial fibrosis in a non-tropical area: from initial presentation to high urgent heart transplantation
Source: BMC Cardiovasc Disord. 2019 Dec 19;19:302. doi: 10.1186/s12872-019-1243-8 (PMC6933894; doi:10.1186/s12872-019-1243-8)

Additional file 6.
Still image of left ventriculography, right anterior oblique (RAO) view
(A) Dashed line depicts original endocardial borders and apical contrast dye sparing due to EMF.

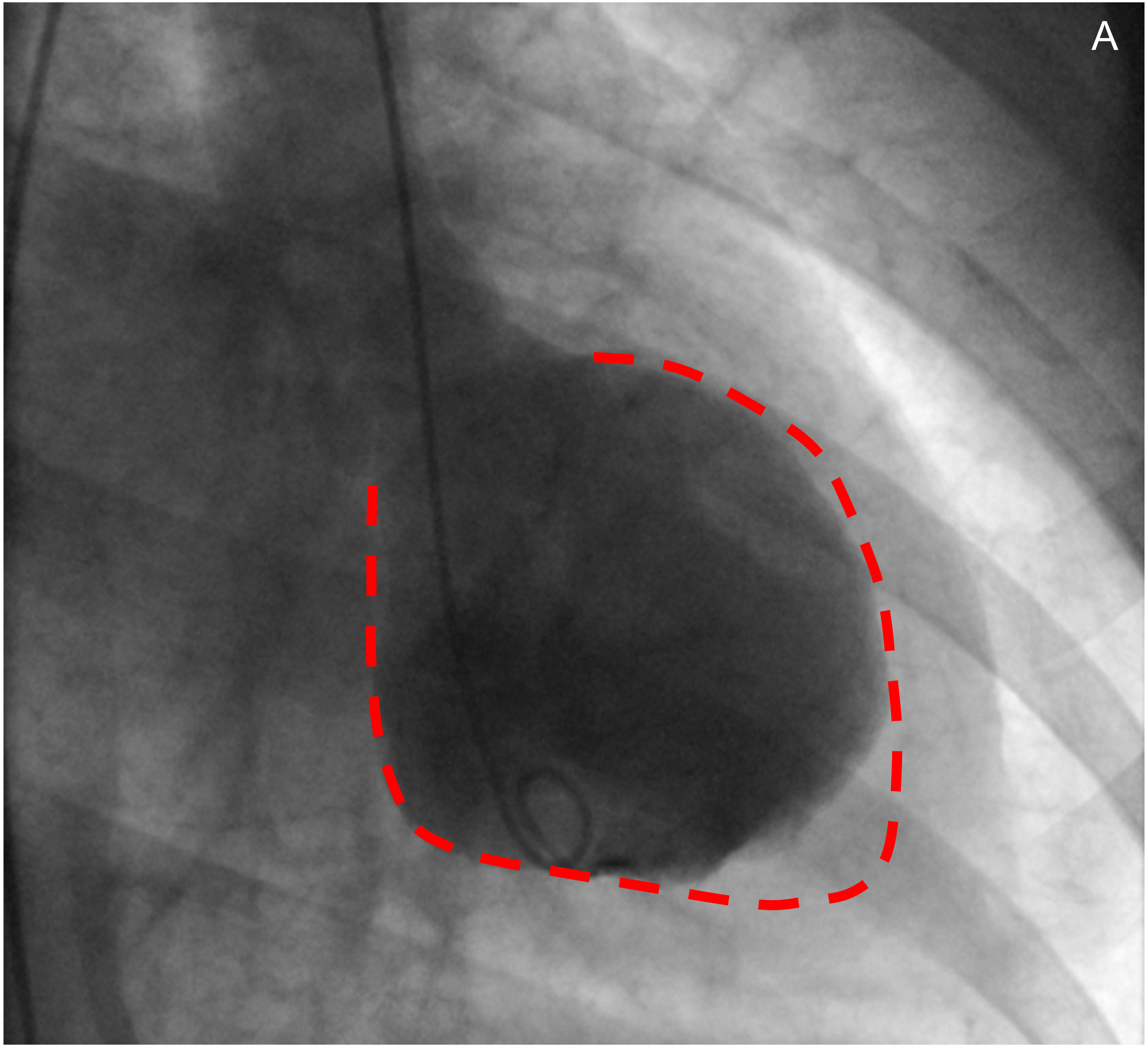

Supplement: Supplementary file 5 — Additional file 5. Still image of left ventriculography, right anterior oblique (RAO) view. [file 12872_2019_1243_MOESM5_ESM.pdf]
